# Patients' Payments

**Published:** 1920-10-02

**Authors:** 


*To the Editor of* The Hospital.

### Patients' Payments.

*To the Editor of* The Hospital.

Sir,—If only the humour of the story were not so frequently mixed with pathos, some amusing anecdotes connected with the inauguration of charges being made for the maintenance of patients in some of our hospitals might be told. Many attempts, some honest, some fraudulent, will doubtless be made to escape payment. One would imagine from some of the excuses made that outside the hospital the patients lived on air. It is quite certain that they would not be satisfied with anything so unsubstantial inside it! I think anyone connected with the administration of a hospital would endorse this statement. At one hospital where the system of payment has just been started many protests are to be heard from parents of children who are inmates of its wards. One of the most specious was, I think, that of a grandmother, who from outward appearances was no supporter of the Pussyfoot campaign; after protesting that she had no means to pay even a small sum towards the maintenance of her granddaughter, she continued, "It is not as if I could earn any money myself. I have not been able to kneel on my knee since the last air-raid!" One could only think that the excessive piety which danger evidently developed had permanently destroyed the flexibility of the knee, which was unused to such exercise. It was difficult to restrain from suggesting that a little cultivated flexibility of the knee rather than the elbow would enable the payment required to be made.—Yours faithfully,

A Hospital Secretary.

